# Systems Model of T Cell Receptor Proximal Signaling Reveals Emergent Ultrasensitivity

**DOI:** 10.1371/journal.pcbi.1003004

**Published:** 2013-03-28

**Authors:** Himadri Mukhopadhyay, Shaun-Paul Cordoba, Philip K. Maini, P. Anton van der Merwe, Omer Dushek

**Affiliations:** 1 Sir William Dunn School of Pathology, University of Oxford, Oxford, Oxfordshire, United Kingdom; 2 Centre for Mathematical Biology, Mathematical Institute, University of Oxford, Oxford, Oxfordshire, United Kingdom; North Carolina State University, United States of America

## Abstract

Receptor phosphorylation is thought to be tightly regulated because phosphorylated receptors initiate signaling cascades leading to cellular activation. The T cell antigen receptor (TCR) on the surface of T cells is phosphorylated by the kinase Lck and dephosphorylated by the phosphatase CD45 on multiple immunoreceptor tyrosine-based activation motifs (ITAMs). Intriguingly, Lck sequentially phosphorylates ITAMs and ZAP-70, a cytosolic kinase, binds to phosphorylated ITAMs with differential affinities. The purpose of multiple ITAMs, their sequential phosphorylation, and the differential ZAP-70 affinities are unknown. Here, we use a systems model to show that this signaling architecture produces emergent ultrasensitivity resulting in switch-like responses at the scale of individual TCRs. Importantly, this switch-like response is an emergent property, so that removal of multiple ITAMs, sequential phosphorylation, or differential affinities abolishes the switch. We propose that highly regulated TCR phosphorylation is achieved by an emergent switch-like response and use the systems model to design novel chimeric antigen receptors for therapy.

## Author Summary

Recognition of antigen by the T cell antigen receptor (TCR) is a central event in the initiation of adaptive immune responses and for this reason the TCR has been extensively studied. Multiple studies performed over the past 20 years have revealed intriguing findings that include the observation that the TCR has multiple phosphorylation sites that are sequentially phosphorylated by the kinase Lck and that ZAP-70, a cytosolic kinase, binds to these sites with different affinities. The purpose of multiple sites, their sequential phosphorylation by Lck, and the differential binding affinities of ZAP-70 are unknown. Using a novel mechanistic model that incorporates a high level of molecular detail, we find, unexpectedly, that all factors are critical for producing ultrasensitivity (switch-like response) and therefore this signaling architecture exhibits systems-level emergent ultrasensitivity. We use the model to study existing therapeutic chimeric antigen receptors and in the design of novel ones. The work also has direct implications to the study of many other immune receptors.

## Introduction

T cells patrol the body in search of infection and cancer derived antigens [1], [2]. They are activated to respond by interactions between T cell antigen receptors (TCRs) on their surface and antigens, in the form of peptides bound to major histocompatibility complexes (pMHCs), on the surfaces of antigen presenting cells (APCs). When activated, T cells can lyse infected cells, secrete cytokines, and perform other effector functions that collectively allow T cells to initiate and regulate adaptive immune responses [1].

The importance of the TCR in initiating and regulating adaptive immune responses has meant that TCR proximal proteins have been extensively studied. The TCR itself is a multi-subunit receptor that contains subunits for ligand binding (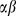 heterodimer) and signal transduction (CD3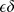 and CD3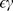 heterodimers, and 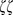 homodimer). Collectively, the signal transducing subunits contain 20 tyrosine residues distributed on 10 immunoreceptor tyrosine based activation motifs (ITAMs) [3]. These ITAMs are phosphorylated by the SRC-family tyrosine kinase Lck and dephosphorylated by the tyrosine phosphatases, such as CD45. Phosphorylated ITAMs serve as docking sites for the tandem SH2 domain-containing cytosolic kinase ZAP-70, which, upon binding, is able to catalyze additional reactions propagating downstream signaling. Despite the small number of molecules involved, their interactions form the basis of a complex regulation network which is poorly understood [4].

**Figure 1:**
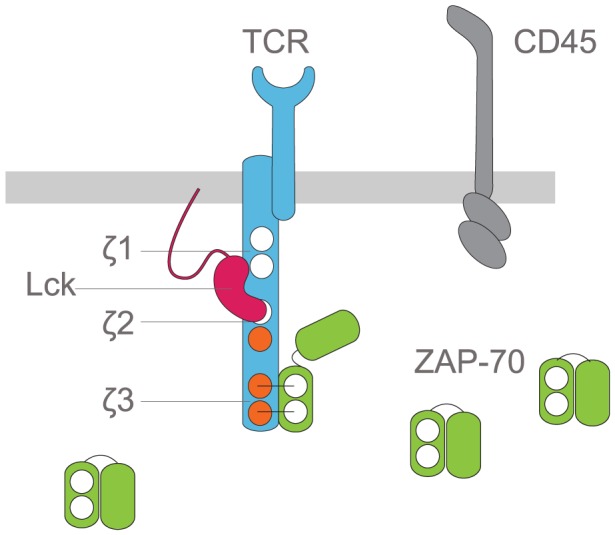
Schematic of the T cell receptor proximal signaling molecules considered in the systems model. We consider the TCR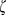-chain containing three ITAMs, labelled as 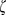
3 (membrane-distal) to 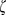
1 (membrane-proximal). These ITAMs are sequentially phosphorylated by the tyrosine kinase Lck and dephosphorylated by the phosphatase CD45. The cytosolic kinase ZAP-70 contains tandem SH2 domains which are able to bind to doubly (fully) phosphorylated ITAMs with differential affinities, with the smallest affinity to 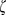
3 and largest affinity to 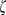
1. When bound to phosphorylated ITAMs, ZAP-70 is able to propagate signaling by phosphorylating downstream signaling molecules and adaptors.

There are many intriguing aspects of this signaling module [4]. While each CD3 signal transducing subunit contains a single ITAM, the 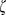-chain contains three ITAMs [3] (Fig. 1). These 3 ITAMs have been shown to be sequentially phosphorylated (membrane-distal to membrane-proximal) by Lck [5] and it has been shown that ZAP-70 binds to these ITAMs with increasing affinities (membrane-distal to membrane-proximal) [6]–[10]. In addition, CD45, a phosphatase that dephosphorylates these ITAMs, is thought to be among the most abundant molecules on T cells [11]. The purpose of multiple ITAMs, their sequential phosphorylation, the differential binding affinities of ZAP-70, and the abundance of CD45 remain unknown.

The binding of pMHC to the TCR is thought to change the kinase-phosphatase ratio leading to phosphorylation of TCR ITAMs and the initiation of an intracellular signaling cascade that leads to T cell activation, which, if inappropriate, can result in autoimmune disorders [12]. Therefore TCR ITAM phosphorylation is thought to be tightly regulated. Given that Lck and CD45 are constitutively active in resting T cells [13], [14], spontaneous (stochastic) pMHC-independent fluctuations in the local Lck or CD45 concentrations can lead to ITAM phosphorylation. These fluctuations may also allow for ITAM phosphorylation in response to endogenous pMHC. On the other hand, maximal ITAM phosphorylation should proceed for antigenic pMHC. Switch-like responses at the scale of individual TCRs provide thresholds, so that phosphorylation is prevented over a wide range of enzyme concentrations below the threshold, and all-or-none responses, so that maximal ITAM phosphorylation proceeds beyond the threshold. However, it is presently unknown whether the TCR proximal signaling architecture supports a switch-like response at the scale of individual TCRs.

In recent years, therapies have been developed to exploit T cells by re-directing them towards infected or cancerous cells [2]. Several research groups have developed therapies based on chimeric antigen receptors (CARs), whereby the variable domains of a monoclonal antibody recognizing pathogen or cancer derived antigens is fused to a signaling chain, most commonly the TCR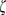-chain [15]. These CARs are transfected into T cells that are then adoptively transferred into patients, generating a pathogen/cancer specific T cell population. However, the antigenic targets of CARs, although highly expressed on cancerous cells are not exclusively expressed on these cells. This observation, coupled with the fact that the extracellular domains of CARs often exhibit very high affinity to their target antigens, means that off-target cell killing can be frequent [15]. Therefore, optimal CARs should exhibit low potency, so that T cells only respond to cells that highly express target antigens, and CARs should also exhibit a high maximum response to ensure complete target cell killing when responding. Although CARs are routinely modified in order to achieve these desired properties, there is presently no mechanistic model to guide the rational development of the signaling domains of CARs to obtain optimal properties.

Here, we construct a systems model of TCR proximal signaling that includes the 3 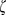-chain ITAMs, their sequential phosphorylation by Lck, and the differential binding affinity of ZAP-70 to these ITAMs. We find that this signaling architecture produces a novel mechanism of ultrasensitivity (switch-like response) at the scale of individual TCRs. Interestingly, we find that all three factors are critical for generating a switch-like response, so that removal of ITAMs, sequential phosphorylation, *or* differential ZAP-70 binding affinities abolishes the switch and therefore this switch can be thought of as an emergent property of the TCR proximal signaling architecture. Our results provide a rationale for the intriguing architecture of TCR proximal signaling and provide a mechanistic framework for the design of novel CARs with desired properties.

## Results

### Multiple TCR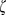-chain ITAMs produce signal amplification, high potency, and ultrasensitivity

We construct a systems model that incorporates the enzymatic kinetics of the tyrosine kinase Lck in sequentially phosphorylating and the dominant phosphatase CD45 in dephosphorylating the three 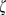-chain ITAMs [5]. In this way, the third membrane-distal ITAM (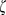
3) must be doubly phosphorylated before Lck is able to phosphorylate the second ITAM (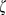
2), and so on. The model incorporates ZAP-70 by allowing it to bind each ITAM when it is doubly phosphorylated, independent of reactions taking place by Lck or CD45 on other ITAMs. The affinity between ZAP-70 and 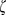
1 is taken to be 10-fold higher than 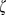
2, which we take to have an affinity 10-fold higher than 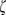
3. The exact affinity values for ZAP-70 binding for each ITAM differ between studies [6]–[10] and therefore we vary these parameters. All enzymatic reactions are modelled in full without any simplifications. The system of ordinary-differential-equations (ODEs) is generated in BioNetGen [16], a rule-based framework for generating biochemical reaction networks, and produced 53 chemical species and 168 reactions (see Methods). The number of chemical species and reactions is large despite only including 4 distinct molecules because the systems model accurately captures all possible molecular complexes that can form.

**Figure 2:**
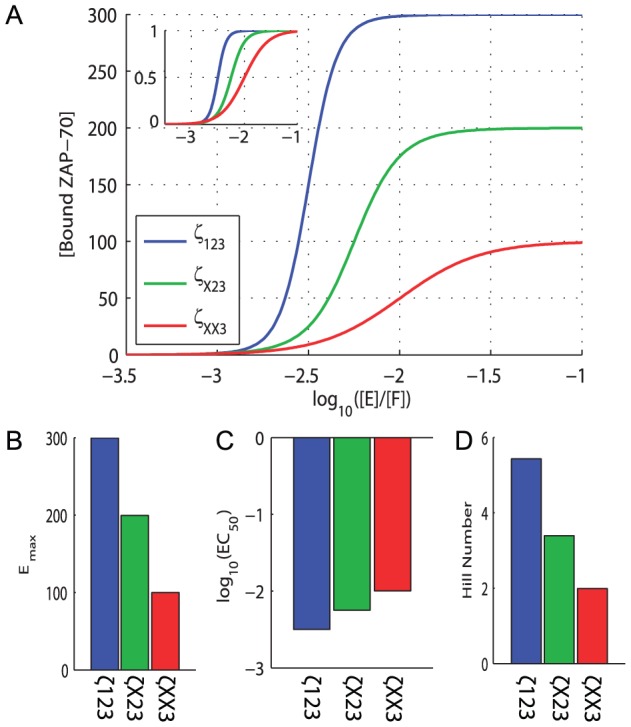
Multiple 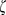-chain ITAMs not only mediate signal amplification, but also increase potency and sensitivity. A) The concentration of ITAM-bound ZAP-70 as a function of the relative kinase (E) to phosphatase (F) concentration. As expected, when the phosphatase is in excess the 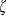-chain is dephosphorylated and ZAP-70 cannot bind whereas when the kinase is in excess the 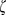-chain is phosphorylated and ZAP-70 is fully bound. Results are shown for the wild-type 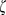-chain (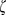
123) and for 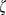-chains where the first and second ITAMs are removed, 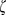
X23 and 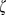
XX3, respectively. Inset shows normalized curves, highlighting differences in potency (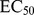) and sensitivity (Hill number), which is a measure of the curve steepness. Each curve is fit to a Hill function to extract estimates of B) the maximum (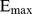), C) the potency (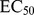), and D) the sensitivity (Hill number). Model and parameter values can be found in Methods.

To study how this signaling architecture regulates the activity of ZAP-70, the concentration of ITAM-bound ZAP-70 was calculated as a function of the relative concentration of active Lck and CD45 (Fig. 2A). In the case of the wild-type TCR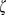-chain (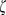
123), we unexpectedly observed an ultrasensitive or switch-like response, whereby small perturbations to Lck or CD45 produced large changes in the concentration of bound ZAP-70. We quantified this observation by fitting a Hill function to the kinase-phosphatase curve to extract estimates of the maximum (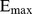, Fig. 2B), the potency (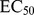, Fig. 2C), and the sensitivity (Hill number, Fig. 1D). We found a Hill number of 5.2, which is greater than 1, indicating a switch-like response. This ultrasensitivity is unexpected because the concentrations of the modifying enzymes (Lck, CD45) are comparable or in excess of the substrate (TCR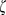-chain) and therefore the observed ultrasensitivity is not a result of the classic zero-order mechanism [17].

We next investigated the contribution of multiple ITAMs. We repeated the calculations when removing the first ITAM (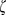
X23) or both the first and second ITAMs (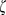
XX3) (Fig. 2A). As expected, we observed a reduction in the maximum concentration of bound ZAP-70 (Fig. 2B), reflecting the signal amplification characteristic of multiple ITAMs [18], [19]. However, removal of ITAMs also decreased potency (Fig. 2C) and sensitivity (Fig. 2D). These effects were not mediated by substrate concentration, as increasing the concentration of 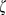
XX3 by a factor of 3 did not restore ultrasensitivity or potency (not shown). Therefore multiple ITAMs on a single 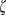-chain are predicted to mediate not only signal amplification, but also potency and sensitivity.

### ZAP-70 binding modulates potency and ultrasensitivity

**Figure 3:**
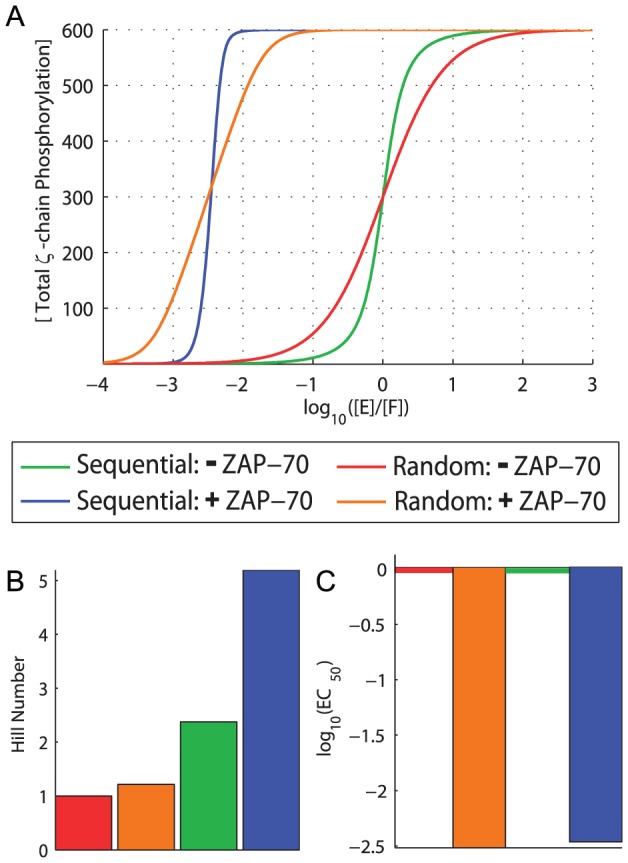
ZAP-70 binding to phosphorylated ITAMs enhances both ultrasensitivity and potency. A) The concentration of total 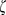-chain phosphorylation as a function of the relative concentration of active kinase (E) to phosphatase (F). Results are shown for sequential phosphorylation (blue, green) and random phosphorylation (red, orange) in the absence (blue, red) and presence (green, orange) of ZAP-70. B) Hill numbers and C) 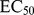 for all four curves reveal that ZAP-70 binding dramatically increases both ultrasensitivity and potency when phosphorylation is sequential but not random.

We next investigated the contribution of ZAP-70 binding to potency and sensitivity. To do this, we calculated the total concentration of phosphorylated 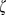-chain, as may be detected by an anti-phosphotyrosine antibody, as a function of the ratio of active enzymes in, respectively, the absence and presence of ZAP-70 (Fig. 3A, blue and green curves). A marked increase in potency and ultrasensitivity is observed in the presence of ZAP-70 (Fig. 3B,C).

**Figure 4:**
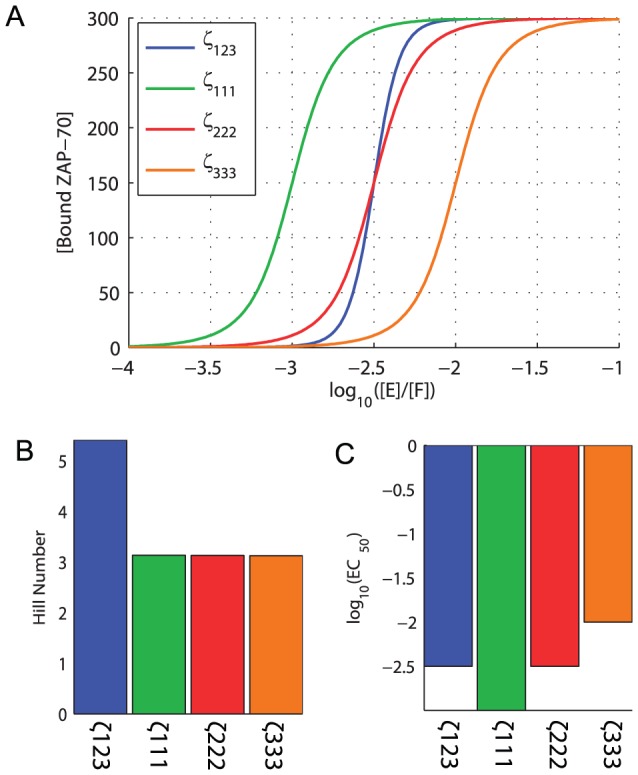
The absolute ZAP-70 affinity for 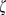-chain ITAMs modulates potency. A) The concentration of bound ZAP-70 as a function of the concentration of active kinase (E) to phosphatase (F). Results are shown for the wild-type 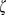-chain (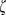
123) and for three additional 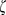-chains that contain all high affinity ITAM 1 (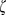
111), intermediate affinity ITAM 2 (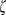
222), or low affinity ITAM 3 (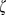
333). Comparison of these 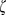-chains reveals that B) sensitivity is unchanged whilst C) potency is modulated.

The increase in potency (decrease in 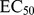) in the presence of ZAP-70 occurs because ITAMs bound by ZAP-70 become inaccessible to phosphatases. Therefore, the absolute ZAP-70 affinity for ITAMs is expected to determine potency. Comparisons between the wild-type 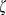-chain (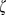
123) and domain duplication constructs that contain three copies of the high affinity ITAM (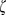
111), intermediate affinity ITAM (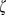
222), and low affinity ITAM (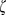
333) reveal that increased potency can be achieved by increasing the ZAP-70 affinity (Fig. 4A–C). The high potency (low 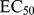) observed in the presence of ZAP-70 provides a plausible explanation for the abundance of CD45 on T cells. In order to keep the TCR unphosphorylated, an excess of phosphatase is required to balance the constitutive activity of Lck and the binding of ZAP-70.

**Figure 5:**
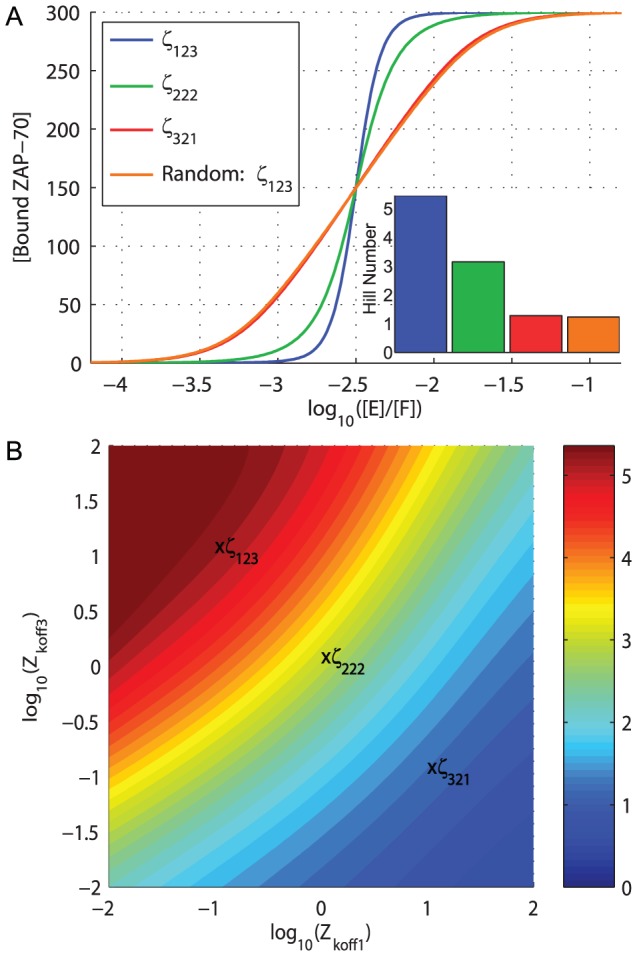
Differential ZAP-70 affinity and sequential phosphorylation produces ultrasensitivity. A) The concentration of bound ZAP-70 as a function of the relative concentration of active kinase (E) to phosphatase (F). Shown are the wild-type 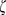-chain (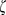
123), and additional constructs where all ITAMs are identical (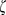
222), switched (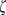
321), or where phosphorylation is no longer sequential (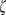
123 Random). The Hill numbers (inset) reveal that ultrasensitivity is decreased if ZAP-70 does not exhibit differential affinity (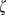
222), if the affinity decreases as the 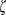 is sequentially phosphorylated (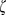
321), or if phosphorylation is no longer sequential. B) Heat map of Hill numbers as a function of the ZAP-70 unbinding rate for ITAM 1 (Z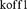) and ITAM 3 (Z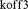), where the unbinding rate for ITAM 2 is fixed at 1 s^−1^. The calculation is performed under sequential phosphorylation. Maximum sensitivity is found in the top left of the heat map, where ZAP-70 binds with the largest affinity to ITAM 1 and with lowest affinity to ITAM 3. The heat map is repeated using an alternate measure of sensitivity in Fig. S3. Note that the heat map colour scheme in panel B is not related to the colour scheme in panel A.

We observed reduced sensitivity for 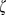-chains containing identical ITAMs (Fig. 4) and therefore surmised that the differential ZAP-70 affinities produce ultrasensitivity. In Fig. 5A we compare the wild-type 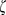-chain (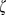
123) to a construct containing identical ITAMs (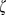
222) and to a construct where ITAM 1 and 3 have switched positions (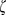
321). We observe that ultrasensitivity relies not only on differential ZAP-70 affinities but also on differential affinities that increase in the direction of phosphorylation (i.e. membrane-distal to membrane-proximal). This is comprehensively illustrated in Fig. 5B, which shows a heat map of Hill numbers as a function of the ZAP-70 unbinding rate for ITAM 1 (x-axis) and ITAM 3 (y-axis) when the unbinding rate for ITAM 2 is fixed at 1 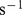. Ultrasensitivity is observed when the unbinding rate is small for ITAM 1 and large for ITAM 3.

The ITAMs located on the TCR 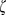-chain are conserved across species [3] yet alignment of the three 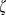-chain ITAMs within a species reveals little homology (Fig. S1). This observation suggests that differences in ITAM sequence are functionally important. Differential binding affinities between ZAP-70 and the 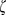-chain ITAMs require differences in ITAM sequence and we have predicted the functional importance of differential ZAP-70 binding affinities. Given that Lck and CD45 also interact with these ITAMs, it is possible that they do so with differential affinities. Differential affinities between the ITAMs and Lck, CD45, or both Lck and CD45 can produce ultrasensitivity in the absence of ZAP-70 (Fig. S2).

In summary, the absolute ZAP-70 affinity for the TCR ITAMs controls potency while the differential ZAP-70 affinity produces ultrasensitivity.

### Sequential phosphorylation is required for ultrasensitivity

The observation that ultrasensitivity relies on ZAP-70 affinities increasing in the direction of sequential phosphorylation (Fig. 5A, 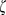
123 vs 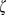
321), suggests that sequential phosphorylation may contribute to ultrasensitivity. We contrasted sequential phosphorylation to random phosphorylation, whereby each ITAM can be independently phosphorylated or dephosphorylated. We found that removal of sequential phosphorylation abolished ultrasensitivity in both total 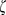-chain phosphorylation (Fig. 3A, 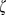
123 Random) and in bound ZAP-70 (Fig. 5A, 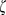
123 Random).

### Ultrasensitivity is an emergent property of the TCR proximal signaling architecture, relying on multiple ITAMs, sequential phosphorylation, and differential ZAP-70 affinity

We have shown the contribution of multiple ITAMs, sequential phosphorylation, and differential ZAP-70 affinity to producing ultrasensitivity. Importantly, ultrasensitivity critically relies on each of these factors so that removal of multiple ITAMs (Fig. 2A, 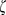
XX3), removal of sequential phosphorylation (Fig. 5A, Random 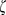
123), *or* differential ZAP-70 affinity (Fig. 5B) can markedly reduce or abolish ultrasensitivity. We therefore conclude that ultrasensitivity is an emergent property of the TCR proximal signaling architecture.

The origin of the proposed emergent ultrasensitivity is in the effective cooperative binding of the ZAP-70 and it can be compared to classical ultrasensitivity observed in allosteric models of Haemoglobin [20]. In these models, as the total ligand concentration increases the concentration of bound ligand exhibits an ultrasensitive response (with large Hill numbers) because ligand binding induces an allosteric conformational change in the receptor that increases the effective binding affinity of subsequent ligand binding. Similarly, increasing the concentration of Lck increases the phosphorylation of the TCR which sequentially produces docking sites for ZAP-70 of increasing affinity. In both cases if the affinity does not increase as molecules bind to the receptor ultrasensitivity is lost. In the case of the TCR, increases in affinity critically rely on the binding sites being sequentially phosphorylated and ZAP-70 having different affinities for these sites, implicitly implying a requirement for multiple ITAMs.

### Design of novel chimeric antigen receptors

As described in the Introduction, chimeric antigen receptors (CARs) are routinely developed without predictive mechanistic models of signaling. We therefore applied the present mechanistic model to understand existing CARs and to design novel CARs. The growing consensus in the community is that optimal CARs should exhibit high affinity to target antigens, low antigen potency, and large maximum responses (see Introduction). Early studies compared CARs containing the TCR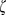-chain and the FC
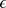
RI-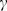-chain [21], [22]. These studies showed improved cytotoxic responses from 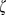-chain fused CARs compared to FC
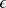
RI-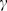-chain fused CARs. The key differences between these two signaling chains are ITAM multiplicity and ZAP-70 affinity; whereas the 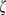-chain contains 3 ITAMs that bind ZAP-70 with various affinities, the FC
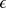
RI-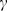-chain contains only a single low affinity ITAM. The FC
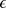
RI-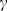 ITAM has an affinity comparable to the low-affinity 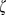
3 ITAM [10].

**Figure 6:**
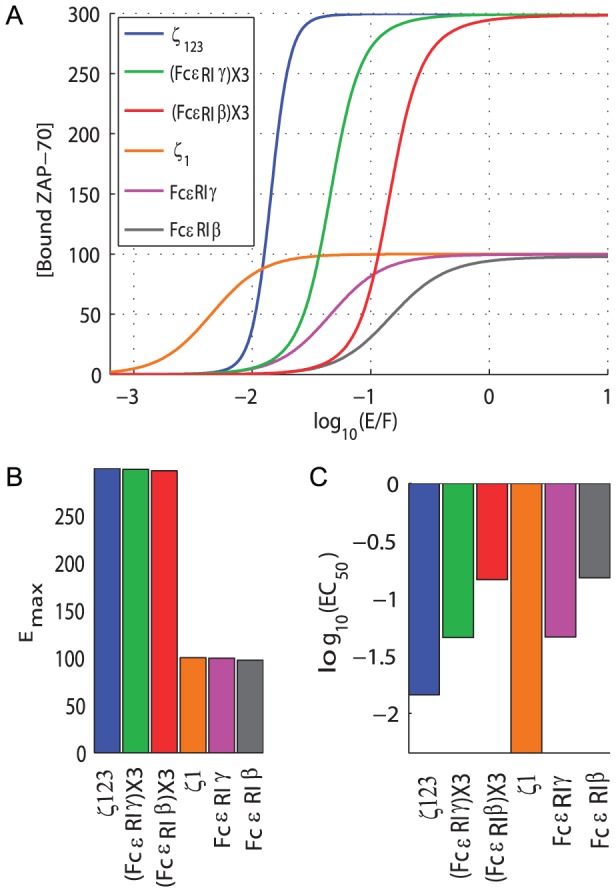
The mathematical model predicts novel chimeric antigen receptors (CARs) design. A) The concentration of bound ZAP-70 as a function of the relative concentration of active kinase (E) to phosphatase (F) for six cytoplasmic CAR domains. These domains include the wild-type 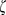-chain (blue), chains containing 3 copies of the low affinity ITAM from FC
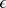
RI-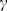 (green) and FC
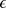
RI-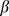 (red), a single high affinity 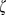-chain ITAM (orange), and chains containing a single copy of the low affinity ITAM from FC
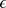
RI-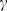 (magenta) and FC
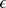
RI-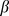 (grey). B-C) Illustrates the 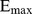 and 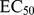 for all CARs shown in panel A. Most CARs are developed based on the wild-type 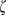-chain but this construct, although having a large 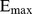 has an undesirably large potency (low 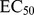). Novel CARs containing multiple low affinity ITAMs (e.g. (FC
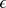
RI-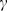)x3, (FC
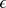
RI-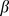)x3) have the desirably low potency (large 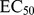) while maintaining the desirably large 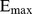.

In Fig. 6 we compare six different CAR designs. CARs containing a single ITAM taken from 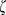
1, FC
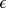
RI-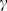, and FC
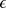
RI-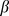 exhibit successively lower ZAP-70 affinity, with 10-fold decreased affinity between 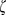
1 and FC
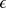
RI-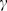 and a further 10-fold decrease in affinity between FC
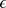
RI-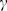 and FC
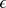
RI-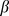 [10]. Using these ITAMs, we construct single or triple ITAM CARs and find that low potency and maximum responses are best achieved by a triple low affinity ITAM signaling chain based on the ITAM from FC
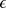
RI-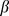.

In summary, although the commonly used TCR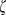-chain exhibits a larger maximum compared to the single ITAM-containing FC
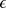
RI-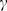, it has an undesirably large potency (small 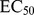). We predict that improved CARs, with reduced off-target cell killing, can be generated by using three (or more) low affinity ITAMs, such as the FC
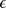
RI-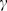 or FC
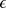
RI-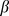 ITAM. Experimental assays to evaluate these novel CARs are discussed below.

## Discussion

We have used a systems model to reveal that multiple ITAMs, sequential phosphorylation, and differential ZAP-70 binding produces emergent switch-like responses at the scale of individual TCRs. This provides a rationale for understanding the functional significance of these previously described aspects of the TCR proximal signaling architecture.

### Differential binding affinity

Evidence for differential ZAP-70 binding affinities comes from competition experiments performed at low temperatures with the SH2 domains of ZAP-70 [6]–[9], [23]. Future work is required to directly measure the affinity of full ZAP-70 at 37°C. Given that switch-like responses can be produced by differential binding of Lck, CD45, or both, (see Fig. S2) it will be interesting to experimentally measure the enzymatic activities of Lck and CD45 with respect to each 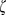-chain ITAM.

### Sequential phosphorylation

We have assumed that phosphorylation of the TCR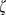-chain proceeds sequentially from the membrane-distal to the membrane-proximal ITAM [5]. However, earlier work suggested an alternate and more complex sequence of phosphorylation whereby individual tyrosines across ITAMs are phosphorylated first, possibly before any individual ITAM is fully phosphorylated [24]. This alternate sequence is compatible with our model provided that fully phosphorylated ITAMs appear in sequence from membrane-distal to membrane-proximal. The mechanism underlying sequential phosphorylation of the TCR 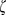-chain is unknown [4]. Recently, it has been proposed that lipid-binding segments of the cytoplasmic domain of PECAM-1 mediate sequential tyrosine phosphorylation [25]. Given that lipid-binding basic residue rich stretch (BRS) are present in the TCR signaling subunits and have been shown to associate with the plasma membrane [26]–[28], it may be that their interactions mediate sequential phosphorylation.

### Significance of high levels of phosphatase

Our detailed model has revealed that an excess of CD45 over Lck is required to keep the TCR 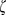-chain dephosphorylated because although Lck and CD45 may have similar enzymatic rates, the binding of the ZAP-70 SH2 domains to phosphorylated ITAMs protects them from dephosphorylation. Previous work has demonstrated that SH2 domains protect tyrosines from dephosphorylatoin by phosphatases [29]. This asymmetry in signaling, whereby molecules bind and protect phosphorylated but not dephosphorylated tyrosines, may apply to other ITAM, ITIM, and ITSM mediated signaling and may explain the need for abundant phosphatases on immune cells. Mathematical models have previously highlighted the need for high phosphatase activity in order to explain the rapid dephosphorylation of tyrosine phosphorylated receptors [30], [31].

### Relation to existing mathematical models

We have studied the regulation of the TCR 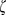-chain using a systems model that includes Lck, CD45, the TCR 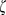-chain, and ZAP-70. Despite including only four molecules, the interaction network that we have generated is very large because of the combinatorial complexity that emerges from including a high level of molecular detail. Previous studies have included these molecules and many others [32], [33] but at the cost of removing molecular detail.

The activity of Lck has been proposed to be regulated by CD45 and several other molecules. A previous mathematical model has predicted that this regulation may produce bistability in the activity of Lck [34] but, to our knowledge, bistability in Lck has not been experimentally observed. Moreover, experimental work has shown that the concentration of Lck in its various phosphorylation states remains largely unchanged upon T cell activation [14]. We have omitted this in the present work because it would dramatically increase the complexity and uncertainty of the model but expect to extend the model as additional information becomes available. We note that the precise mechanism by which antigen binding to the T cell receptor alters the kinase-phosphatase balance leading to receptor phosphorylation, a process termed receptor triggering, remains controversial [12]. A complete understanding of receptor triggering will provide insights into how antigens of varying affinity alter TCR proximal signaling [1], [35], [36].

The TCR 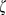-chain contains 6 phosphorylation sites and is therefore considered a multisite substrate. There is a growing body of literature investigating the purpose of multisite phosphorylation [37]. Previously, it was proposed that membrane-anchored, but not cytosolic, multisite substrates can produce ultrasensitivity [38]. However, to achieve Hill numbers of greater than 5 membrane-anchored substrates require 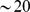 phosphorylation sites whereas the TCR 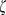-chain is predicted to achieve this cooperativity with only 6 sites. This level of cooperativity is achieved by both ZAP-70 binding and sequential phosphorylation. The binding of ZAP-70 is a form of product inhibition, which has previously been proposed to increase cooperativity [39], [40] and sequential phosphorylation has also been implicated in switch-like responses [41]. Although multiple mechanisms of ultrasensitivity have been previously proposed, the TCR 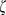-chain utilizes mechanisms that cooperate so that large ultrasensitivity is achieved with only 6 phosphorylation sites. We note that the ultrasensitivity we have reported is distinct from multi-stability, which has been previously proposed to be taking place on multisite substrates [39], [42].

### Implications for other receptors

In addition to the TCR, other immune receptors (e.g. Fc
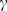
RIII, IGSF4 [43], NKp30, NKp46) associate with the 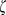-chain and therefore the predictions made here may be applicable to these receptors. There are many other receptors, termed Non-catalytic Tyrosine-Phosphorylated Receptors (NTRs) [44], with multiple phosphorylation sites that are regulated by extrinsic enzymes and like the TCR, spurious activation of these receptors may be avoided by switch-like responses that contain strong thresholds. The mathematical model used in the present study can be used to study the regulation of NTR phosphorylation.

### Implications for CARs

CARs are a class of therapeutic receptors that contain variable domains of a monoclonal antibody that recognizes pathogen or cancer derived antigens fused to the TCR 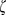-chain [2], [15]. The majority of CARs rely on the TCR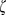-chain, or variants thereof, because early studies showed that CARs containing the TCR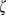-chain exhibited higher target cell killing efficiencies compared to other signaling chains, such as the FC
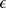
RI-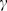 [22]. In contrast to the 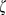-chain, the FC
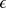
RI-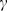 contains only a single ITAM that binds ZAP-70 with low affinity, comparable to the low affinity interaction between ZAP-70 and 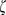
3 [10]. This improved killing efficiency is likely due to ITAM multiplicity on the 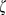-chain (Fig. 5, 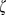
123 vs. FC
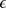
RI-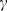). However, the 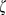-chain containing CAR also has the unfavourable property of increased potency. As shown in Fig. 5, novel CAR designs that maintain low potency and high maximum are possible by using three copies of low affinity ITAMs taken from either FC
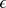
RI-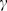 or FC
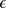
RI-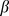.

We propose that *in vitro* target cell killing assays can be used to determine optimal CARs. However, these assays need to be performed using titration of antigens on the target cells (with a fixed number of target cells) not the more commonly performed assay that involves titrating the number of target cells (with a fixed antigen concentration). Optimal CARs should exhibit low potency (large 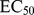), only killing cells that highly express specific antigens, and large maximum (large 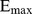), killing many cells that highly express specific antigens. An alternative assay is to use measures of cellular activation (e.g. expression of CD107a) in response to titrations of immobilized antigen. In the present work we predict that CARs fused to wild-type 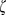-chain or signaling chains that contain three copies of low affinity ITAMs, such as the FC
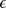
RI-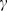 or FC
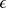
RI-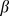 ITAMs, will exhibit similar maxima. However, only the signaling chains containing three copies of the low affinity ITAMs will exhibit the desired low potency (large 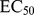).

## Methods

### Systems model

The model includes the TCR 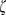-chain with 6 phosphorylation sites (distributed on 3 ITAMs) that are sequentially phosphorylated by Lck (membrane-distal to membrane-proximal) and sequentially dephosphorylated by CD45. We assume that Lck and CD45 cannot simultaneously bind the TCR 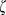-chain. We take 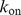, 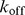, and 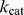 to be identical for both enzymes in the main text but discuss alterations in these parameters in Fig. S2. Doubly phosphorylated ITAMs serve as binding sites for ZAP-70 (provided that CD45 is not bound to the ITAM) and therefore a maximum of three ZAP-70 can bind per TCR 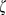-chain. Binding of ZAP-70 to an ITAM occurs independently of reactions on other ITAMs and while bound, ZAP-70 protects the ITAM from dephosphorylation by CD45 [29]. Note that ZAP-70 binding only relies on doubly phosphorylated ITAMs and binding is not restricted to any particular sequence. Differential ZAP-70 affinity is implemented by decreasing the 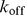 for ITAM binding by a factor of 10 between each ITAM (membrane-distal to membrane-proximal) while keeping 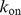 identical between ITAMs. Alterations in 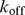 between ITAMs can be found in Fig. 5B.

The system of ODEs representing the biochemical reaction network based on the above scheme was generated in BioNetGen [16] (see [38], [45]–[47] for additional examples of the application of BioNetGen to model reaction networks) and integrated in Matlab (Mathworks, MA). The BioNetGen file used to generate this network is available in Text S1 and contains the set of default parameters (10 reaction rates and 4 chemical concentrations). The parameter values used for concentrations and reaction rates are estimated based on known values. However, we stress that our main results are not sensitive to the precise values of the parameters. The concentration of ITAM-bound ZAP-70 or total 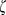-chain phosphorylation was calculated at steady-state by varying the concentration of phosphatase (identical results can be obtained by varying the concentration of the kinase). Total 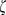-chain phosphorylation is the multiplicative product of 1) the concentration of a particular TCR 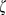 isoform (regardless of whether it is bound to an enzyme) and 2) the number of phosphate groups attached to the isoform.

In addition to the wild-type signaling architecture, several modified architectures were investigated. We compared sequential to random phosphorylation by allowing Lck and CD45 to catalyze reactions at ITAMs independent of the phosphorylation state of the 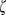-chain. This random or unstructured phosphorylation scheme generated 2688 reactions and over 500 chemical species compared to 168 reactions and 53 chemical species generated for the sequential scheme. Note that a mixed scheme, whereby phosphorylation is sequential but dephosphorylation is random cannot be implemented without an excessive number of additional assumptions (e.g. if dephosphorylation takes place on ITAM 3 whilst ITAM 2 is fully phosphorylated, is is unclear if sequential phosphorylation requires re-phosphorylation of ITAM 3 before phosphorylation on ITAM 1 can take place). The various 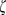-chain constructs were implemented by changing 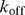 between ZAP-70 and each ITAM. For example, the 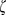
222 construct is generated by taking 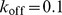 s^−1^ between ZAP-70 and all 3 ITAMs. In each modified architecture, we preserve all remaining properties of the wild type architecture.

### Curve fitting

The profiles of ITAM-bound ZAP-70 and total TCR 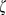-chain phosphorylation are fit to logarithmic Hill Functions using *lsqcurvefit* in Matlab (Mathworks, MA),
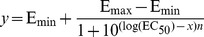
where 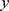 corresponds to either ITAM-bound ZAP-70 or total TCR 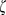-chain phosphorylation and 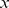 the kinase-to-phosphatase ratio on a logarithmic (base 10) scale. The fitted parameter values include: 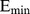 and 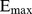, which correspond to the minimum and maximum values, respectively, 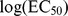, which is the logarithmic value of the kinase-to-phosphatase ratio that yields half of the maximal response (otherwise known as potency), and 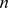, which is the Hill number that determines the steepness of the response (otherwise known as the sensitivity).

## Supporting Information

Figure S1Alignment of the TCR 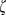-chain. A) An alignment of the full 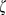-chain across multiple species reveals a high degree of conservation of all three ITAMs. B) An alignment of the three 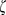-chain ITAMs in humans reveals that with the exception of the defining features of the ITAM motif itself, there is very little sequence homology between the three ITAMs. Given the conservation across species, this suggests a functional role for differences between 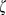-chain ITAMs within a species.(EPS)

Figure S2Ultrasensitivity in the absence of ZAP-70 can be mediated by differential affinities of Lck, CD45, or both. A) The total phosphorylation of TCR 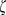-chain is shown as a function of the concentration of active Lck to CD45 in the absence of ZAP-70. When both Lck and CD45 have identical ITAM affinities or put another way, when there are no differential affinities, we observe a small Hill number (none, orange curve). Ultrasensitivity is improved if Lck, CD45, or both have differential ITAM affinities (red, green, blue curves, respectively). As for ZAP-70, the differential affinity is achieved by keeping the on-rate identical but varying the off-rate by 10-fold between each ITAM. The catalytic rate is identical between each ITAM. B) Hill numbers for each case in panel A. Note that the increased ultrasensitivity relies on the Lck affinity to increase from membrane-distal to membrane-proximal ITAM while the CD45 affinity must increase from membrane-proximal to membrane-distal, which we have assumed to be the case.(EPS)

Figure S3An alternate measure of sensitivity for the heat map in Fig. 5B. We repeat the calculations in Fig. 5B except display the ratio of EC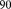 to EC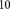 on a log-scale (i.e. log
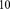
(EC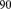/EC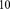)) instead of the Hill numbers. We find identical trends and can therefore conclude that the Hill number is a reasonable measure of sensitivity.(EPS)

Text S1 BioNetGen source code used to generate the wild-type systems model of T cell receptor proximal signaling.
(PDF)
