# Supplementary material for: Systems Model of T Cell Receptor Proximal Signaling Reveals Emergent Ultrasensitivity
Source: PLoS Comput Biol. 2013 Mar 28;9(3):e1003004. doi: 10.1371/journal.pcbi.1003004 (PMC3610635; doi:10.1371/journal.pcbi.1003004)
Supplement: Text S1 — BioNetGen source code used to generate the wild-type systems model of T cell receptor proximal signaling. (PDF) [file pcbi.1003004.s004.pdf]

Text S1
BioNetGen source code used to generate the wild-type systems model of T cell receptor proximal signaling.

```
#Enzymatic modifications of TCR zeta and ZAP-70 binding to fully phosphorylated ITAMs.

begin parameters

#Kinase-substrate on rates
ek_on 1e-4

#Kinase-substrate off rates
ek_off 10.0

#Kinase catalysis rates
ek_cat 10.0

#Phosphatase-substrate on rates
fk_on 1e-4

#Phosphatase-substrate off rates
fk_off 10.0

#Phosphatase catalysis rates
fk_cat 10.0

#Binding parameters for ZAP-70 binding
zap_on 1.0

zap_off1  10
zap_off2   1
zap_off3 0.1

#Total substrate (S_T), kinase (E_T), phosphatase (F_T), and ZAP-70 (Z_T) concentrations
E_T 100
F_T 100
S_T 100
Z_T 5000

end parameters

begin molecule types

#Substrate
#Attribute b~1 indicates that an enzyme is bound to substrate and b~0 indicates no enzyme is bound
#Attributes Y1, Y2, and Y3 each refer to individual ITAMs.
#Each ITAM can be unphosphorylated (Y~U), phosphorylated on one tyrosine (Y~P), or doubly phosphorylated (Y~2P)

S(b~0~1,Y1~U~P~2P,Y2~U~P~2P,Y3~U~P~2P)

#Kinase
E(b)

#Phosphatase
F(b)

#ZAP-70
Z(b)

end molecule types

begin seed species

S(b~0,Y1~U,Y2~U,Y3~U) S_T
E(b) E_T
F(b) F_T
Z(b) Z_T

end seed species
```

```
begin reaction rules

#ITAM1 MODIFICATIONS

E(b) + S(b~0,Y1~U,Y2~U,Y3~U) <-> E(b!1).S(b~1,Y1~U!1,Y2~U,Y3~U) ek_on, ek_off
E(b!1).S(b~1,Y1~U!1,Y2~U,Y3~U) -> E(b) + S(b~0,Y1~P,Y2~U,Y3~U) ek_cat

F(b) + S(b~0,Y1~P,Y2~U,Y3~U) <-> F(b!1).S(b~1,Y1~P!1,Y2~U,Y3~U) fk_on, fk_off
F(b!1).S(b~1,Y1~P!1,Y2~U,Y3~U) -> F(b) + S(b~0,Y1~U,Y2~U,Y3~U) fk_cat

E(b) + S(b~0,Y1~P,Y2~U,Y3~U) <-> E(b!1).S(b~1,Y1~P!1,Y2~U,Y3~U) ek_on, ek_off
E(b!1).S(b~1,Y1~P!1,Y2~U,Y3~U) -> E(b) + S(b~0,Y1~2P,Y2~U,Y3~U) ek_cat

F(b) + S(b~0,Y1~2P,Y2~U,Y3~U) <-> F(b!1).S(b~1,Y1~2P!1,Y2~U,Y3~U) fk_on, fk_off
F(b!1).S(b~1,Y1~2P!1,Y2~U,Y3~U) -> F(b) + S(b~0,Y1~P,Y2~U,Y3~U) fk_cat

#ITAM2 MODIFICATIONS

E(b) + S(b~0,Y1~2P!?,Y2~U,Y3~U) <-> E(b!1).S(b~1,Y1~2P!?,Y2~U!1,Y3~U) ek_on, ek_off
E(b!1).S(b~1,Y1~2P!?,Y2~U!1,Y3~U) -> E(b) + S(b~0,Y1~2P!?,Y2~P,Y3~U) ek_cat

F(b) + S(b~0,Y1~2P!?,Y2~P,Y3~U) <-> F(b!1).S(b~1,Y1~2P!?,Y2~P!1,Y3~U) fk_on, fk_off
F(b!1).S(b~1,Y1~2P!?,Y2~P!1,Y3~U) -> F(b) + S(b~0,Y1~2P!?,Y2~U,Y3~U) fk_cat

E(b) + S(b~0,Y1~2P!?,Y2~P,Y3~U) <-> E(b!1).S(b~1,Y1~2P!?,Y2~P!1,Y3~U) ek_on, ek_off
E(b!1).S(b~1,Y1~2P!?,Y2~P!1,Y3~U) -> E(b) + S(b~0,Y1~2P!?,Y2~2P,Y3~U) ek_cat

F(b) + S(b~0,Y1~2P!?,Y2~2P,Y3~U) <-> F(b!1).S(b~1,Y1~2P!?,Y2~2P!1,Y3~U) fk_on, fk_off
F(b!1).S(b~1,Y1~2P!?,Y2~2P!1,Y3~U) -> F(b) + S(b~0,Y1~2P!?,Y2~P,Y3~U) fk_cat

#ITAM3 MODIFICATIONS

E(b) + S(b~0,Y1~2P!?,Y2~2P!?,Y3~U) <-> E(b!1).S(b~1,Y1~2P!?,Y2~2P!?,Y3~U!1) ek_on, ek_off
E(b!1).S(b~1,Y1~2P!?,Y2~2P!?,Y3~U!1) -> E(b) + S(b~0,Y1~2P!?,Y2~2P!?,Y3~P)  ek_cat

F(b) + S(b~0,Y1~2P!?,Y2~2P!?,Y3~P) <-> F(b!1).S(b~1,Y1~2P!?,Y2~2P!?,Y3~P!1) fk_on, fk_off
F(b!1).S(b~1,Y1~2P!?,Y2~2P!?,Y3~P!1) -> F(b) + S(b~0,Y1~2P!?,Y2~2P!?,Y3~U) fk_cat

E(b) + S(b~0,Y1~2P!?,Y2~2P!?,Y3~P) <-> E(b!1).S(b~1,Y1~2P!?,Y2~2P!?,Y3~P!1) ek_on, ek_off
E(b!1).S(b~1,Y1~2P!?,Y2~2P!?,Y3~P!1) -> E(b) + S(b~0,Y1~2P!?,Y2~2P!?,Y3~2P) ek_cat

F(b) + S(b~0,Y1~2P!?,Y2~2P!?,Y3~2P) <-> F(b!1).S(b~1,Y1~2P!?,Y2~2P!?,Y3~2P!1) fk_on, fk_off
F(b!1).S(b~1,Y1~2P!?,Y2~2P!?,Y3~2P!1) -> F(b) + S(b~0,Y1~2P!?,Y2~2P!?,Y3~P) fk_cat

#ZAP70 BINDING

Z(b) + S(Y1~2P) <-> Z(b!1).S(Y1~2P!1) zap_on, zap_off1

Z(b) + S(Y2~2P) <-> Z(b!1).S(Y2~2P!1) zap_on, zap_off2

Z(b) + S(Y3~2P) <-> Z(b!1).S(Y3~2P!1) zap_on, zap_off3

end reaction rules

begin observables

1 Molecules Bound_ZAP Z(b!+)

2 Molecules Szero S(b~?,Y1~U!?,Y2~U!?,Y3~U!?)

3 Molecules Sone S(b~?,Y1~P!?,Y2~U!?,Y3~U!?)

4 Molecules Stwo S(b~?,Y1~2P!?,Y2~U!?,Y3~U!?)

5 Molecules Sthree S(b~?,Y1~2P!?,Y2~P!?,Y3~U!?)

6 Molecules Sfour S(b~?,Y1~2P!?,Y2~2P!?,Y3~U!?)

7 Molecules Sfive S(b~?,Y1~2P!?,Y2~2P!?,Y3~P!?)

8 Molecules Ssix S(b~?,Y1~2P!?,Y2~2P!?,Y3~2P!?)

end observables

generate_network({overwrite=>1});

writeMfile({});
```
